# Reversion of glucocorticoid-induced senescence and collagen synthesis decrease by LY294002 is mediated through p38 in skin

**DOI:** 10.7150/ijbs.73915

**Published:** 2022-10-18

**Authors:** Quoc-Vu Le, Su-Ying Wen, Chih-Jung Chen, Chih-Yang Huang, Wei-Wen Kuo

**Affiliations:** 1 Department of Biological Science and Technology, College of Life Sciences, China Medical University, Taichung 406, Taiwan, ROC.; 2 Ph.D. Program for Biotechnology Industry, China Medical University, Taichung 406, Taiwan, ROC.; 3 Department of Dermatology, Taipei City Hospital, Renai Branch, Taipei, Taiwan, ROC.; 4 Center for General Education, Mackay Junior College of Medicine, Nursing, and Management, Taipei, Taiwan, ROC.; 5 Division of Breast Surgery, Department of Surgery, China Medical University Hospital, Taichung 40447, Taiwan, ROC.; 6 Cardiovascular and Mitochondrial Related Disease Research Center, Hualien Tzu Chi Hospital, Buddhist Tzu Chi Medical Foundation, Hualien 970, Taiwan, ROC.; 7 Center of General Education, Buddhist Tzu Chi Medical Foundation, Tzu Chi University of Science and Technology, Hualien 970, Taiwan, ROC.; 8 Department of Medical Research, China Medical University Hospital, China Medical University, Taichung 404, Taiwan, ROC.; 9 Graduate Institute of Biomedical Sciences, China Medical University, Taichung 404, Taiwan, ROC.; 10 Department of Medical Laboratory Science and Biotechnology, Asia University, Taichung 413, Taiwan, ROC.

**Keywords:** glucocorticoid, clobetasol propionate, LY294002, skin atrophy, p38 MAPK, dermal fibroblasts

## Abstract

Glucocorticoids (GCs) are the most common treatment for inflammatory skin disorders; however, they show several adverse side effects, including atrophy and collagen decrease following chronic treatment. In particular, transcription factors and p38 signaling for collagen synthesis have been shown to be suppressed by the active glucocorticoid receptor (GR). LY294002 (LY), a phosphoinositide 3-kinase (PI3K) inhibitor, has been reported to protect keratinocytes in epidermis against GC-induced hypoplasia; however, its protective effect in dermis remains unclear. Furthermore, clobetasol propionate (CP) is the most used commercial synthetic GC, yet studies on how CP causes side effects in dermal fibroblasts are limited. In this study, dermal atrophy was modeled using CP in human dermal fibroblasts (HDFs) and C57BL/6 mice. CP treatment significantly upregulated FK506 binding protein 5 (FKBP51), an atrophy marker (2.4 ± 0.25 and 3.3 ± 0.3 fold in *in vitro* and *in vivo*, respectively), phosphorylated GR (1.96 ± 0.08 and 2.29 ± 0.25 fold in *in vitro* and *in vivo*, respectively), decreased fibroblast proliferation (82.71 ± 1.95% in *in vitro*), reduced collagen synthesis (0.36 ± 0.05 and 0.3 ± 0.1 fold in *in vitro* and *in vivo*, respectively), and induced aging, all of which were reversed by LY treatment (from 1.43 ± 0.08 to 2.8 ± 0.12 fold) without showing growth inhibition and exerting the anti-inflammation of CP. Interestingly, the protective effect of LY was dose-dependently reversed by treatment with a p38 inhibitor and reached 2.9 ± 0.15 fold at dose 20 µM. Taken together, our results demonstrate that LY reduced CP-induced upregulation of the atrophy marker FKBP51, GR phosphorylation, and GR nuclear translocation via the activation of p38, whilst maintaining the anti-inflammatory effect of glucocorticoids.

## Introduction

Glucocorticoids (GCs) and mineralocorticoids, classified as corticosteroids, are steroid hormones secreted by the adrenal cortex which is regulated by the hypothalamic-pituitary-adrenal axis. Corticosteroids are generally used to refer to GCs. GCs are pivotal regulators of a wide variety of fundamental processes such as metabolism, cell proliferation, differentiation, and inflammation 1, 2. Owing to their lipophilic structure, GCs are capable of promptly diffusing through cellular membranes to exert their biological functions. Interestingly, many studies have corroborated that dermal cells possess a complete biochemical response to efficient steroidogenesis 3. The first synthetic GC was introduced to the clinic in the late 1950s and it was developed as an effective medicine for topical and systemic therapeutics of inflammatory and autoimmune viscera classified into atopic dermatitis, including eczema, psoriasis, and sclerosis 4. Unfortunately, chronic intervention with GCs may result in various adverse effects including osteoporosis, muscle waste, and skin atrophy 5, 6.

The effects of GCs are mediated by the glucocorticoid receptor (GR), a transcription factor. Under balanced homeostasis, GR binds to co-chaperones comprising heat shock protein 90 and immunophilins to form a united complex in the cytoplasm. Along with hormone stimulation, GR is activated by phosphorylation to translocate into the nucleus, where it positively (transactivation, TA) or negatively (transrepression, TR) regulates gene expression. TA is triggered mainly by the binding of GR homodimers to glucocorticoid-responsive elements (GRE) in gene promoters and enhancers. TR is mediated by different mechanisms, including negative protein-protein interactions between GR and other TFs such as proinflammatory factors NF-κB and AP-1, collagen synthesis component TGF-β1, and p-Smad 2/3 7, 8. Unfortunately, the TR of GCs on TFs for collagen synthesis genes, responsible for extracellular matrix (ECM) organization of dermal fibroblasts, is a particular phenomenon observed in GC-induced skin atrophy.

Skin atrophy is one of the most serious side effects in patients with eczema, psoriasis, and sclerosis after long-term treatment with topical corticosteroids. Chronic GC treatment may result in severe hypoplasia of all skin partitions, increased frailty, laceration, contusions, and defects in skin protective functions 9. FKBP51 (FK506 binding protein 5) is a central driver of steroid-induced skin atrophy of the epidermis, especially in keratinocytes. FKBP51 is also an immunophilin that acts as a co-chaperone of the GR complex for nuclear translocation. The inhibition of FKBP51 has been shown to inhibit the nuclear translocation of GCs and attenuate the glucocorticoid-induced decrease in the proliferation of keratinocytes in the epidermis 9, 10.

Furthermore, skin atrophy related to the dermis results from a disorder of collagen synthesis 11. Transforming growth factor beta (TGF-β) is a ubiquitous and potent cytokine that positively regulates collagen synthesis in the human skin dermis 12. TGF-β binds to specific serine and threonine kinase receptor complexes, including TGF-β receptor type I and II (TβRI and TβRII), triggering phosphorylation of p-Smad2/3. The activation of p-Smad2/3 requires interaction with Smad4 to regulate its translocation into the nucleus. Once in the nucleus, p-Smad2/3 modulates the transcription of target genes, including collagen type I and III, via the Smad-binding element (SBE) promoter 13. Mounting evidence has shown that elevated intrinsic aging, caused by increased ROS production, and extrinsic aging, following exposure to UV radiation and chemicals, contribute to TGF-β/Smad signaling pathway impairment. In turn, the deficiency of TGF-β/Smad signaling results in reduced collagen synthesis in fibroblasts, causing disruption of dermal homeostasis 14, 15. Mammalian MAP kinases (MAPK), including p38, c-jun-N-terminal kinase (JNK), and extracellular signal-regulated kinases (ERKs), regulate collagen synthesis in numerous experimental systems 16. In particular, ERKs and p38 MAPK have been shown to cooperate with the TGF-β pathway in the arrangement of the ECM and turnover 17. In dermal fibroblasts, the wound healing process requires phosphorylation of p38 MAPK, which is expressed along the wound edge and is involved in collagen recovery from wounds 18, 19. Additionally, the inhibition of p38 MAPK also increases apoptosis in damaged dermal fibroblasts, resulting in collagen degradation 20. Therefore, it has been suggested that p38 MAPK possesses a positive role in dermal collagen synthesis.

Several studies have established that the inhibitory actions of rapamycin and phosphoinositide-3-kinase (PI3K) inhibitors on FKBP51 expression can protect keratinocytes against GC-induced atrophy 9, 10. However, it is understood that GC-induced skin atrophy occurs not only in the epidermis, but also in the dermis where dermal fibroblasts contribute majorly to the organization via collagen synthesis. Phenotypic atrophy of the dermis is characteristically related to a decrease in the quantity of collagen and accelerated aging 11. Hence, in this study, we aimed to investigate whether the PI3K inhibitor LY294002 suppresses FKBP51 expression and modulates the translocation of GR to alleviate collagen decrease in HDFs via p38 MAPK.

## Materials & methods

### Cell culture and treatment

Human dermal fibroblasts (HFDs) were purchased from the Bioresource Collection and Research Center (BCRC, Hsinchu, Taiwan). HFDs were cultured in Dulbecco's modified Eagle's medium (DMEM) containing 10% FBS (HyClone), antibiotics (100 μg/mL of penicillin and 100 μg/mL of streptomycin) and incubated in 10% humidified air and 5% CO_2_ at 37°C. To evaluate the protective effect of LY against CP-induced adverse effects in HDFs, HFDs were pre-treated with a 15 μM dose of LY for 6 h and then treated with 0.1 μM of CP (c8037, Sigma-Aldrich) for 24 h. Passages <15 was used as young HFDs in this study.

### Experimental animals and treatment protocol

Seven-week-old male mice, 5 animals per group, were topically applied with 200 μL of vehicle alone (40% isopropyl alcohol) or LY294002 (15 µM/mouse) in same vehicle for 5 h, and then treated with 200 μL clobetasol propionate (CP) in vehicle control (1 μg/mouse) to the back skin once every other day for 4 weeks. For the reverse of LY effect, mice were treated with 40µM of SB203580 in same vehicle before LY treatment 1 h. Epidermis, dermis and hypodermis fat were isolated from the dorsal skin mechanically by scraping. To evaluate the anti-inflammatory effect of CP, mouse ears were pretreated with either vehicle or LY294002 (15 µM/ear), 5 h prior to treatment croton oil (CO) application, 20 μL of 40% solution in isopropyl alcohol, then CP (1 μg/ear) was applied 1 h prior to CO. Animals were sacrificed to harvest ears after 9 h of CO application. Ear swelling defined for inflammation was measured by weighing 5 mm ear punches.

### Antibodies and reagents

The following antibodies were used in this study: FKBP51 (ab12671, Abcam), GR (sc-393232, Santa Cruz), p-GR (#4161, Cell Signaling), IκBα (sc-1643, Santa Cruz) IL-1β (sc-7884, Santa Cruz), IL-6 (sc-130326, Santa Cruz), MKK3 (#5674, Cell Signaling), p-MKK3/6 (#9231, Cell Signaling), MMP1 (sc-21731, Santa Cruz), NF-kB (sc-109, Santa Cruz), p-NF-kB (sc-136548, Santa Cruz), TGF-β1 (sc-31609, Santa Cruz), p-Smad2/3 (sc-11769, Santa Cruz), Sirt1 (ab189494, Abcam) Smad4 (sc-7966, Santa Cruz), COL1A1 (sc-28657, Santa Cruz), COL3A1 (sc-271249, Santa Cruz), p16 (10883-1-AP, proteintech), p21 (sc-6246, Santa Cruz), p53 (1C12) (#2524, Cell Signaling), β-gal (sc-19119, Santa Cruz), MMP-1 (sc-21731, Santa Cruz), β-actin (sc-47778, Santa Cruz), and GAPDH (sc-32233, Santa Cruz). Secondary antibodies (anti-rabbit, anti-mouse, and anti-goat) were purchased from Santa Cruz Biotechnology.

### Transfection

HDFs were transfected with the NF-κB luciferase reporter plasmid using PureFection Reagent (System Biosciences, Palo Alto, CA, USA) according to the manufacturer's instructions and incubated in 10% humidified air and 5% CO_2_ at 37 °C for 24 h after CP or LY treatment for further detection.

### Cell viability assay

Viability was determined by MTT (3-(4,5-dimethylthiazol-2-yl)-2,5-diphenyl tetrazolium bromide) assay. HDFs were inoculated into 96-well plates and incubated with different concentrations of CP or LY for 24 h for dose-dependent detection, or 15 µM of LY, and then co-incubated with 0.1 µM CP for 24 h. After 24 h incubation, the culture was changed to 500 μL of MTT solution 0.5 mg/mL. Following 3 h of incubation at 37 °C, MTT solution was removed and the purified formazan was solubilized in 500 μL of dimethyl sulfoxide (DMSO). Optical density was measured at 570 nm using a spectrophotometer (Bio-Rad, USA).

### Western blot analysis

HDFs were cultured on a 10 cm dish for 24 h for different interventions. To isolate total protein, the culture was washed with cold PBS and resuspended in RIPA lysis buffer (89900, Thermo Fisher Scientific). After incubation for 30 min on ice, the suspension was centrifuged at 12000 rpm for 30 min at 4 °C, and the pellet was discarded. Protein concentration was measured following the protocol of Bio-Rad protein assay dye reagent concentrate (#5000006). Equal concentrations of samples (40 μg) were loaded onto an SDS-PAGE gel and transferred onto PVDF membranes (Millipore, Bedford, Massachusetts, USA). Proteins on the membrane were blocked with buffer (Bullet Blocking One, Nacalai Tesque) for 5 min at room temperature and then washed with PBST. Membranes were incubated with primary antibodies and shaken at 4 °C overnight. Following primary antibody incubation, membranes were incubated with secondary antibodies (anti-rabbit, anti-mouse, or anti-goat IgG).

### Luciferase reporter assay

HDFs were grown in 6 cm cell dishes and co-transfected with the NF-κB luciferase reporter plasmid. After 48 h, the culture was collected and lysed according to the manufacturer's protocol (Promega), and relative luciferase activity was determined using a Dual-Glo Luciferase Assay System (Promega, Sunnyvale, CA, USA).

### Senescence-associated β-galactosidase (SA-β-gal) staining

Senescence of HDFs was measured to assess the ratio of SA-Gal-positive cells. HDFs were seeded in 4-well chamber slide for 24 h for different interventions. HDFs were fixed in 4% paraformaldehyde at 4 °C for 30 min. Cells were washed four times with ice-cold PBS followed by overnight incubation with X-Gal (pH 6.0) at 37 °C. After rinsed at least thrice, cells were photographed under a light microscope.

### Indirect immunofluorescence

HDFs grown in 4-well chamber slides were fixed and permeabilized in 4% paraformaldehyde and 0.1% Triton X-100 for 15 min at room temperature, respectively, prior to incubation of indicated antibodies. The cells were then washed and stained with Alexa Fluor 488 goat anti-rabbit IgG secondary antibody (Cat#A11008, Invitrogen). Images were captured using Olympus CKX53 confocal spectral microscope. The images were processed using the Adobe Photoshop software.

### Extraction of nuclear protein

Cells were grown in 10 cm cell culture dishes and harvested with cold PBS after indicated interventions. Nuclear protein was followed an instruction of Nuclear/Cytosol Fractionation Kit (BioVision, CA, USA) manufacturer. The nuclear protein was quantified by Bradford assay for western blot analysis.

### Histological analysis

Skin tissues from each group were fixed in 4% paraformaldehyde and embedded in paraffin wax. The tissue sections were cut into two-micron thick sections and then counterstained with hematoxylin-eosin (H&E) stain to observe skin thickness, Masson's trichrome (M&T) stain to assess collagen fiber, and immunohistochemistry stain to confirm signaling protein expression in cell model 21. Images of the samples were obtained using Olympus CKX53 microscope (Olympus, Tokyo Japan).

### Statistical analysis

All experiments were conducted at least thrice. Statistical analyses were performed by one-way ANOVA and Student's *t*-test using SigmaPlot 11.0 (SPSS Inc, Chicago, Illinois, USA). Mean and standard deviation shown as mean ± SD were calculated using Microsoft Excel, corresponding to three or more replicates. A p-value below 0.05 was considered significant difference.

## Results

### Clobetasol propionate (CP) dose-dependently induces senescence and collagen synthesis decrease in human dermal fibroblasts (HDFs)

**Figure 1:**
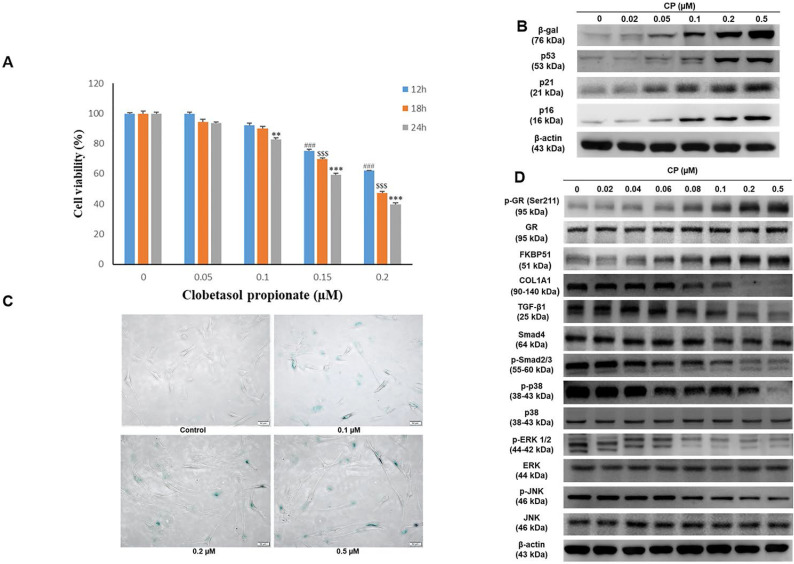
Clobetasol propionate (CP) dose-dependently induces senescence and collagen synthesis decrease in human dermal fibroblasts (HDFs). Cells were treated with CP at doses as indicated for 12, 18, or 24 h. **(A)** Cell viability was detected using the MTT assay. **(B)** The expressions of β-gal and p53 were evaluated by western blot analysis. **(C)** Cell senescence was identified by β-galactosidase staining. **(D)** The expressions of atrophic marker (FKBP51, GR), collagen synthesis pathway (COL1A1, TGF- β1, Smad4, p- smad2/3), and MAPKs (p38, ERK, JNK) were evaluated by western blot analysis. Values shown are means ± SE. Quantification of the results is shown (n = 3), ^#^p< 0.05, ^##^p < 0.01, ^###^p < 0.001 versus control of 12 h; ^$^p< 0.05, ^$$^p < 0.01, ^$$$^p < 0.001 versus control of 18 h; *p < 0.05, **p < 0.01, ***p < 0.001 versus control of 24 h. β-actin was used as a loading control.

GCs have been reported to decrease cell proliferation. A previous study revealed that FKBP51 expression responded to GC treatment 10. In the present study, we confirmed cell viability following CP exposure using the MTT assay. The results demonstrated that cell number decreased in a dose and time-dependent manner at doses ranging from 0-0.2 μM CP (Fig. 1A). Moreover, dramatic elevation of atrophy markers, FKBP51 and GR, as well as aging markers, β-gal and p53, were observed in HDFs after CP exposure. Additionally, collagen synthesis and MAPK signaling decreased in a dose-dependent manner (Fig. 1B-1D). Despite convincing advantages in clinical intervention, there is increasing evidence that GCs can induce senescence via the activation of the p53/p21 and p16 pathways, leading to cell cycle arrest 22. Consequently, collagen fibrils are diminished resulting in a phenotype of both skin aging and atrophy 10, 23. In agreement with previous studies, our results implied that GCs could induce skin aging and impair collagen synthesis. In particular, after 24 h of CP treatment with the indicated doses from 0-0.2 µM, a gradual increase in β-gal and p53 expression examined by western blot was observed, followed by a remarkable upward trend at 0.1 and 0.2 µM (Fig. 1B). Interestingly, the cell senescence condition evaluated by β-galactosidase staining was consistent with the results of the aging marker expression (Fig. 1C). Simultaneously, CP was shown to negatively affect the collagen synthesis pathway. The expression of COL1A1 as well as its upstream signals, including TGF-β1, Smad4, p-Smad2/3, dramatically decreased at doses over 0.1 µM CP (Fig. 1D). Based on these results, doses over 0.1 µM can induce side effects and therefore 0.1 µM CP was selected in the following experiments.

### Dose selection of LY without growth and collagen synthesis inhibition in HDFs

**Figure 2:**
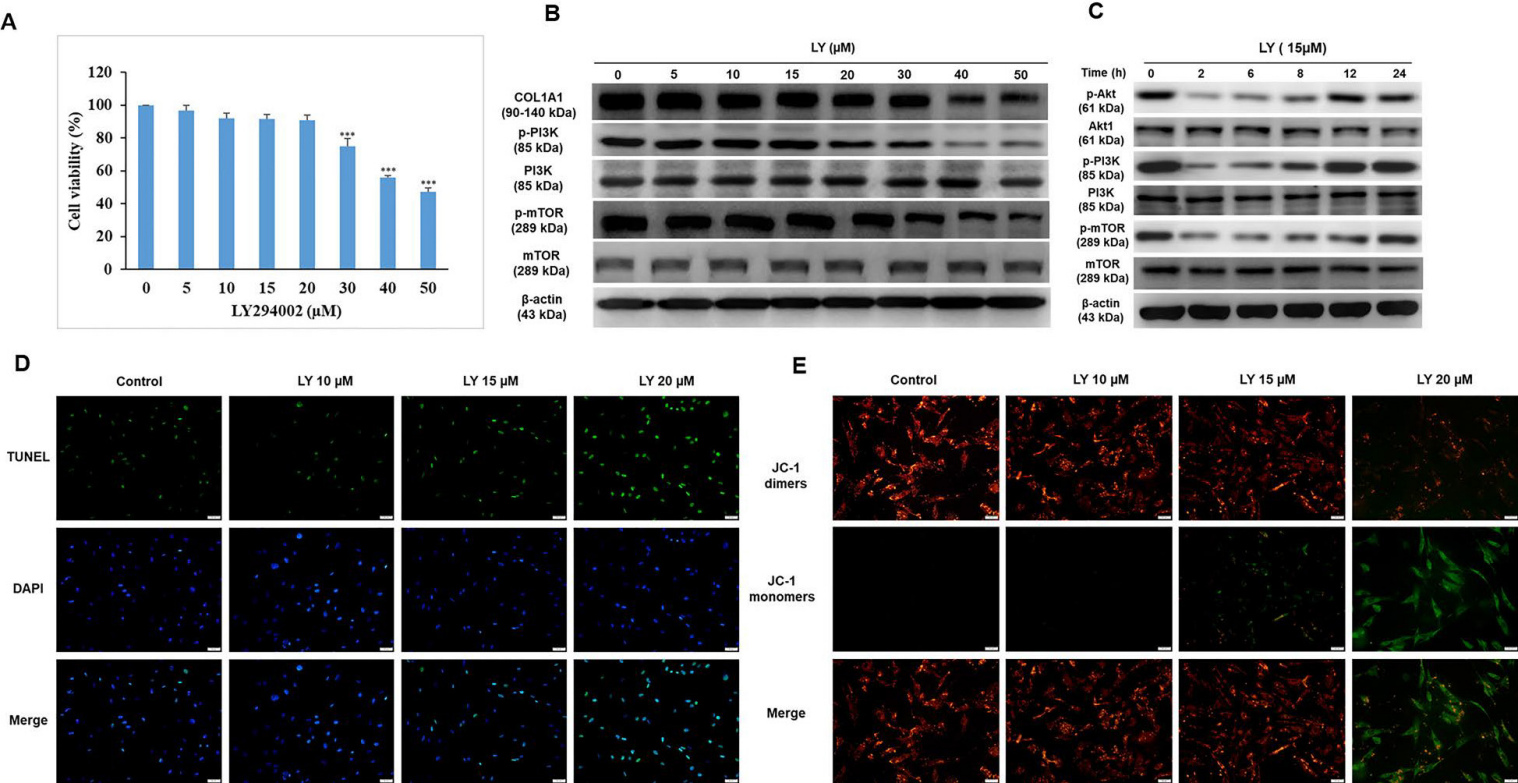
Dose selection of LY294002 (LY) without growth and collagen synthesis inhibition in HDFs. Cells were treated with LY at doses as indicated for 24 h. **(A)** Cell viability was detected using MTT assay. Values shown are means ± SE. Quantification of the results is shown (n = 3). *p < 0.05, **p < 0.01, ***p < 0.001 versus control of 24 h. **(B, C)** The expression of Akt, COL1A1, TGF- β1, mTOR, and PI3K was evaluated by western blot analysis. β-actin was used as a loading control. **(D, E)** Apoptotic condition and mitochondrial membrane potential were analyzed by TUNEL and JC-1 staining assay, respectively.

Although LY has been recently reported to protect keratinocytes against GC-induced atrophy 10, it was originally developed as an anticancer drug. Among the PI3K inhibitors LY is a unique drug that inhibits mTOR signaling, such as rapamycin, and has been approved by the US Food and Drug Administration (FDA) for immunosuppressive and antiproliferative treatments for several diseases, such as tuberous sclerosis, psoriasis, and malignancy 24. In this study, we investigated the applicable dose of LY for HDFs. After treatment with LY (0-50 µM) for 24 h, the results of the MTT assay indicated that the lethal dose of 30-40 µM significantly reduced cell survival (Fig. 2A). To confirm whether LY could inhibit mTOR signaling, the expression of mTOR, p-mTOR, PI3K, and p-PI3K was evaluated using western blotting. The results showed that the levels of p-mTOR and p-PI3K were maintained at 5-15 µM LY, but declined considerably at doses over 20 µM. There was no change in mTOR and PI3K. Furthermore, we observed that the reduced PI3K/Akt/mTOR signaling gradually recovered in a time-dependent manner in the presence of LY at a dose of 15 µM (Fig. 2C). At higher doses of LY (0-15 µM), COL1A1 levels were stabilized (Fig. 2B). Cellular apoptosis and mitochondrial function were confirmed using TUNEL and JC-1 staining assays, respectively (Fig. 2D, 2E).

### The selected dose of LY attenuates GR-induced senescence and collagen synthesis decrease in HDFs

**Figure 3:**
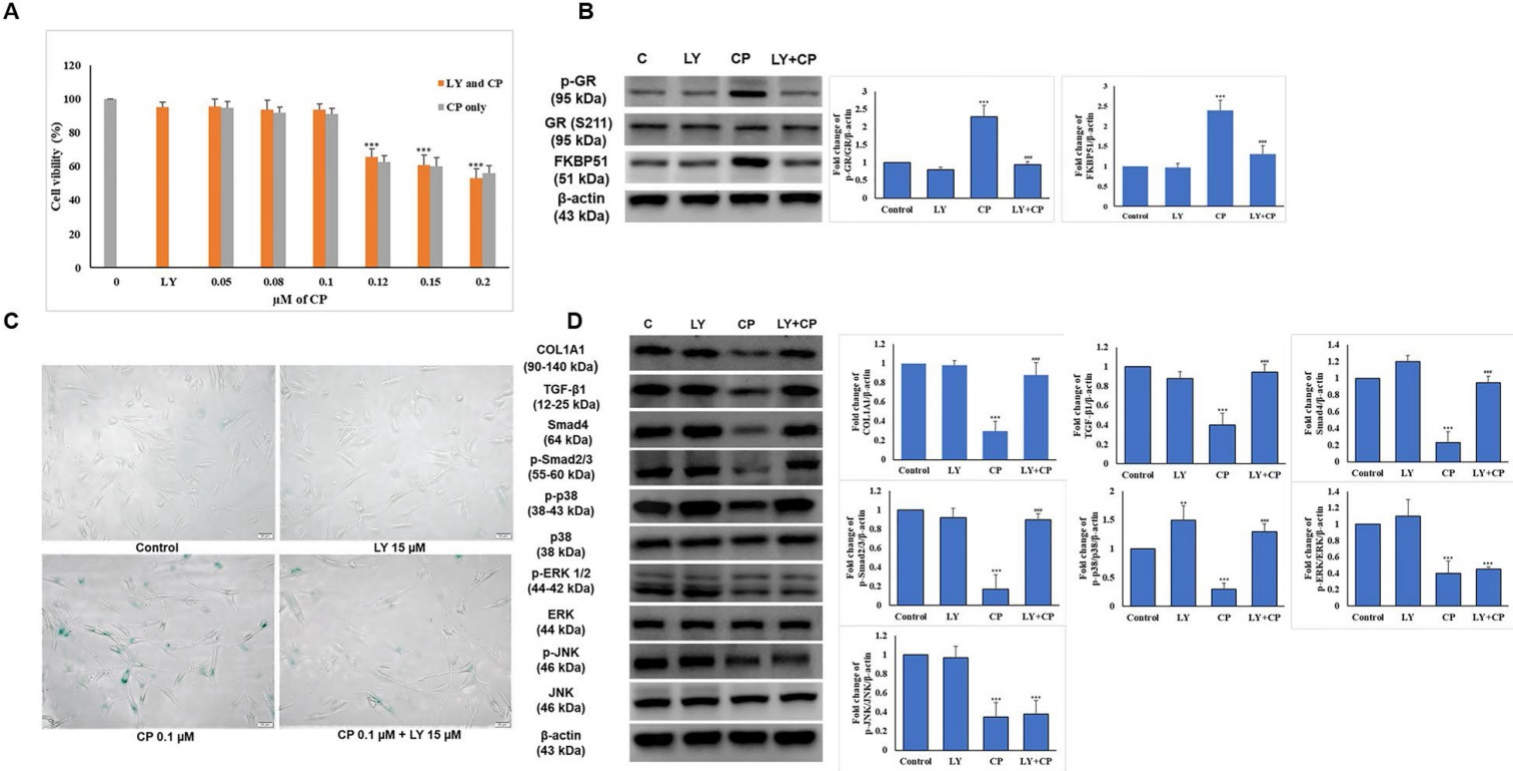
The selected dose of LY attenuates GC-induced senescence and collagen synthesis decrease in HDFs. Cells were pretreated with LY at doses as indicated for 6 h, then treated with 0.1 µM CP for 24 h. **(A)** Cell viability was detected using the MTT assay. **(B)** The expressions of p-GR, GR, and FKBP51 were evaluated by western blot analysis. β-actin was used as a loading control. **(C)** Cell senescence was identified by β-galactosidase staining. **(D)** Components of collagen synthesis, and MAPK signaling pathway in HDFs were identified by western blot. Values shown are means ± SE. Quantification of the results is shown (n = 3), *p < 0.05, **p < 0.01, ***p < 0.001 versus control, ^#^p< 0.05, ^##^p < 0.01, ^###^p < 0.001 versus CP. β-actin was used as a loading control.

To determine whether the combination of CP and LY at the selected dose displays the expected benefit, we examined cell proliferation in addition to the level of GR complex expression using the MTT assay and western blot, respectively. As shown in Fig. 3A, combined with increasing doses of CP (0-0.2 µM), 15 µM LY had no impact on HDFs survival, and reversed the elevation of p-GR and FKBP51 (Fig. 3B) indicating that the dose selection of 15 µM LY and 0.1 µM CP were appropriate for further experiments.

HDFs were treated with 15 µM of LY for 6 h, then the medium was changed to medium 0.1 µM CP followed by incubation for 24 h. The condition of the aging cells was tested with β-galactosidase staining and we observed that LY could reduce cell senescence induced by CP (Fig. 3C). The reduced collagen levels and related signaling by CP were reversed following LY treatment. Despite similar reduced MAPK signaling by CP, p38, but not ERK and JNK, activation was reversed following LY treatment (Fig. 3D). This finding indicates that LY could inhibit the side effects of GCs and thus restore collagen production.

### LY reduces GR activity to modulate its nuclear translocation and reverse collagen synthesis signaling in HDF exposed to GCs

**Figure 4:**
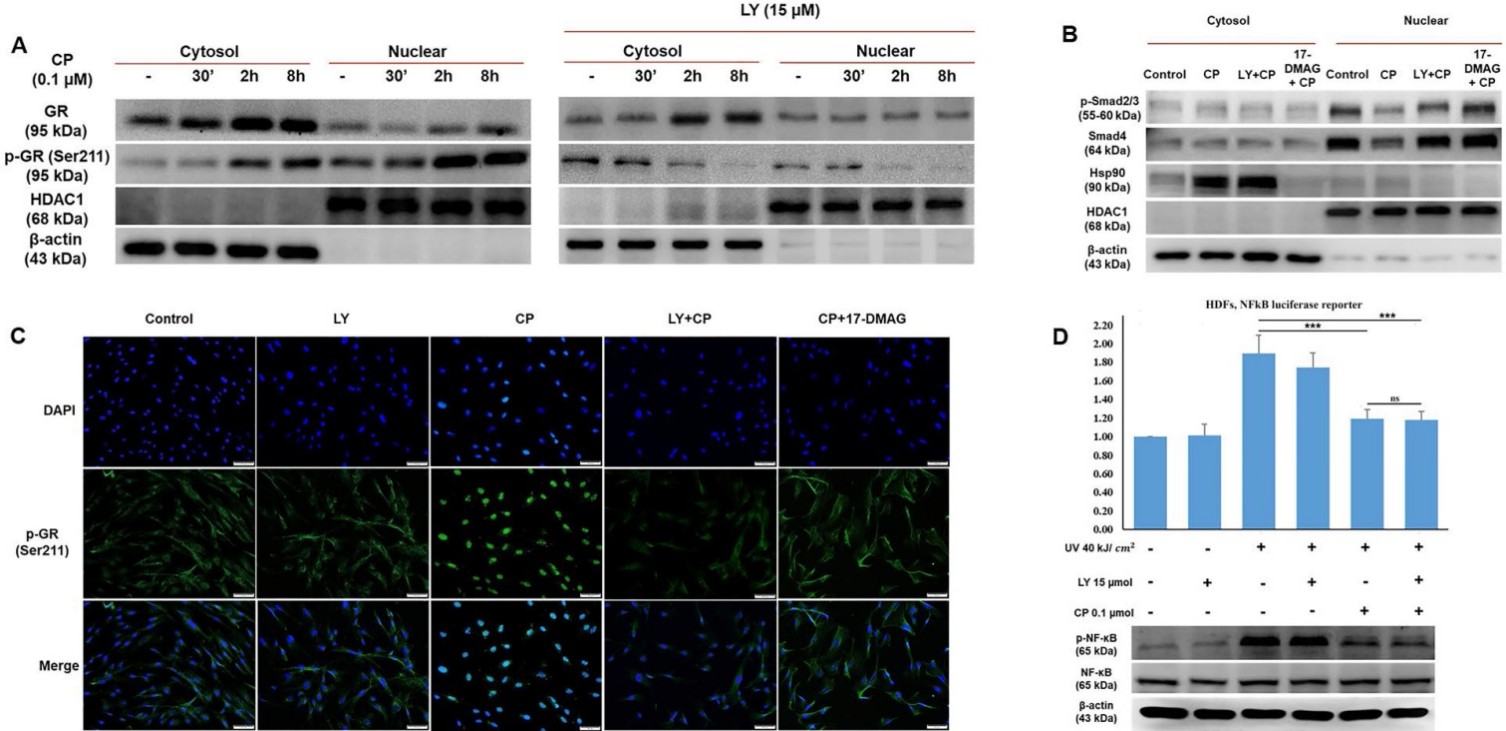
The selected dose of LY prevents GR transactivation resulting in the stability of transcription factors for collagen synthesis without blocking the anti-inflammatory action of GCs. Cells were pre-treated with 15 µM LY for 6 h, then 0.1 µM CP for the indicated time. **(A & B)** The cytoplasmic and nuclear proteins were extracted separately. The expressions of p- GR, GR, Smad4, p-Smad2/3, and Hsp90 were evaluated by western blot analysis. β-actin and HDAC1 were used as loading controls. **(C)** The cellular distribution of p-GR was detected by immunofluorescence. **(D)** Cells transfected with plasmid containing the NF-kB-dependent promoter were exposed 40 kJ/ cm2 of UVB, then pre-treated with 15 µM LY for 6 h, followed by treatment with 0.1 µM CP for 24 h. The activity of NF-kB was analyzed by luciferase reporter. The expressions of p-NF-kB and NF-kB were evaluated by western blot analysis. 17-DMAG was used as an Hsp90 inhibitor. β-actin was used as a loading control. Values shown are means ± SE. Quantification of the results is shown (n = 3), *p < 0.05, **p < 0.01, ***p < 0.001, ns: not significant versus control and CP.

GCs bind to their receptors, undergo phosphorylation, and then exert transactivation or transrepression. It has been documented that phosphorylation plays a vital role in GR activity, especially nuclear translocation 25. To explore in more detail how LY could blunt GR activity, we separately extracted cytosolic and nuclear proteins to identify the different concentrations of the original and phosphorylation form at Ser211 of GR. As shown in Fig. 4A, the results demonstrated that following CP treatment, GR expression considerably increased only in the cytoplasm, whereas p-GR was time-dependently expressed in both the cytoplasm and nucleus after CP treatment. Our findings indicated that the decrease in transcription factors for collagen synthesis, evidenced by the inhibition of nuclear localization induced by CP, was reversed by LY intervention (Fig. 3C). Furthermore, the reduced levels of p-Smad2/3 and Smad4 induced by CP were reversed by LY treatment in nuclear fraction, implying that LY could protect collagen transcription factors from GCs by inhibiting GR translocation, Additionally, 17-DMAG, an inhibitor of heat shock protein 90 (Hsp90), which is a required co-chaperone of GR for ligand binding, activation and translocation, was used as positive control (Fig. 4B). Additionally, inhibition of Hsp90 by 17-DMAG, but not LY, can abolish the anti-inflammatory effect of GC (Supplementary Fig. 2). Next, p-GR was chosen to further detect the inhibitory effect of LY using an immunofluorescence assay. As anticipated, it was clearly observed that during CP treatment, p-GR translocated to the nucleus and pretreatment with LY prevented this phenomenon (Fig. 4C). Taken together, our findings imply that the phosphorylation form of GR at Ser211 can translocate into the nucleus under GC treatment, but the intervention with LY inhibited p-GR nuclear translocation and accumulation.

### LY maintains the anti-inflammatory effect of glucocorticoid on HDFs exposed to UV radiation

Based on our data, the side effects of CP were reversed by treatment with LY, indicating that LY modulated GR activity (Fig. 4A-C). Next, we checked whether LY hampered the anti-inflammatory effects of glucocorticoids. HDFs were transfected with a plasmid containing the NF-κB-dependent promoter for the luciferase reporter and inflammation was induced by UVB at 40 kJ/cm^2^. The results of the luciferase assay showed that increased NF-κB activity by UV exposure could be reduced by CP, with or without LY treatment, indicating that combination treatment with LY still maintained the TR effect of CP on NF-κB activation (Fig. 4D). Simultaneously, western blot data revealed that the level of p-NF-κB in the CP group with and without LY was not different (Fig. 4E). Therefore, although LY could decrease GR activity, the anti-inflammatory effect of GCs remained.

### The involvement of p38 MAPK in the protective effect of LY in HDFs exposed to CP

**Figure 5:**
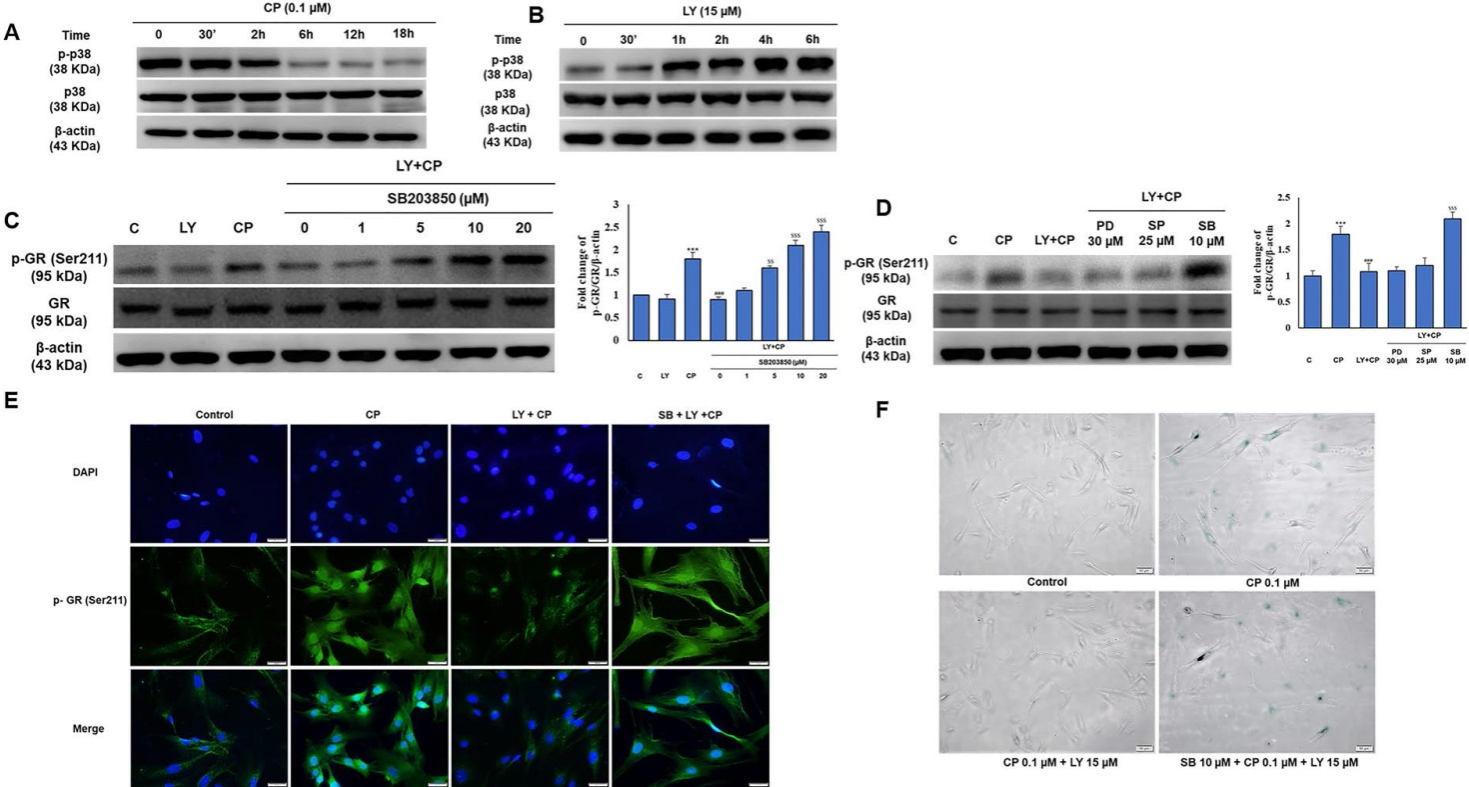
p38 MAPK is involved in the GR modulation by LY in GC treated HDFs. Cells were pre-treated with the indicated doses of MAPK inhibitors (PD98059; ERK inhibitor, SB203580; p38 MAPK inhibitor, or SP600125; JNK inhibitor) followed by LY treatment followed by CP treatment. **(A & B)** The level of p38 MAPK was investigated in a time-dependent manner with CP and LY treatment as evaluated by western blot. **(C & D)** The expression of p-GR and GR were evaluated by western blot. **(E & F)** The cellular distribution of p-GR and senescence were performed by immunofluorescence of p-GR and β-galactosidase staining, respectively. β-actin was used as a loading control. Values shown are means ± SE. Quantification of the results is shown (n = 3), *p < 0.05, **p < 0.01, ***p < 0.001 versus control, ^#^p< 0.05, ^##^p < 0.01, ^###^p < 0.001 versus LY plus CP treatment group.

Several studies have demonstrated that p38 is required for collagen synthesis. As shown in Figure 3C, among the MAPKs protein family members, only the activity of p38 was improved by LY against CP exposure, and we then investigated whether p38 plays a role in LY-reduced GR activation. Interestingly, there was a contrasting trend in p38 time-dependent activity between CP and LY treatment alone (Fig. 5A-B). We confirmed that LY prevented nuclear translocation of GR using pharmacological inhibitors of ERK, p38, and JNK. Western blotting and immunofluorescence data analysis showed that only p38 MAPK was involved in the inhibitory effect of LY on CP-induced GR activation (Fig. 5C-E). Similar results showed that the inhibition of p38 reversed the protective effect of LY against CP-induced HDFs senescence, as detected by β-galactosidase staining (Fig. 5F), indicating that the inhibitory effect of LY on CP-induced GR activation and senescence is mediated through p38 activation.

### LY spares mouse skin from CP-induced atrophy through the mediation of p38 without blocking CP's anti-inflammatory effects of GCs

**Figure 6:**
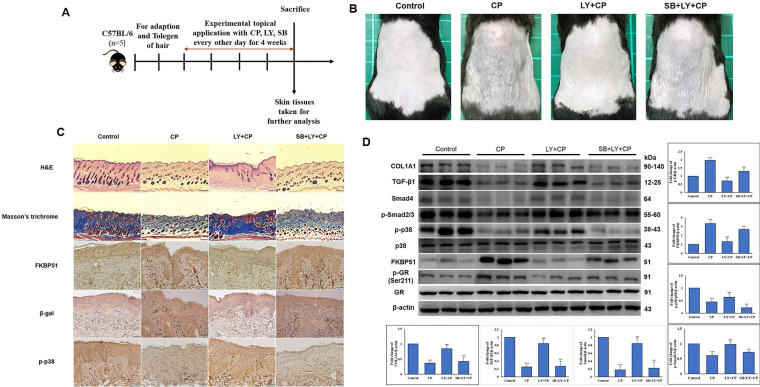
LY spares mouse skin from CP-induced atrophy through the mediation of p38 without blocking CP's anti-inflammatory effects of GC. **(A)** The detailed animal protocol is described in material and method section. Mice were treated with vehicle or CP (1 µg/mouse) with or without LY (50 µM/mouse) followed by SB treatment. **(B)** Photograph depicting atrophy formation on the dorsal skin of mice due to CP exposure. **(C)** The thickness of epidermal and dermal layers was evaluated by H&E staining; Masson's trichrome staining showed the collagen deposition in the dermis. Collagen fibers appear blue and stained immunohistochemically for FKBP51, p-p38, and β-gal. **(D)** The protein levels of collagen synthesis signaling components, atrophic marker, and p38 MAPK in dorsal skin tissues were detected by western blotting. β-actin was used as a loading control. Values shown are means ± SE. Quantification of the results is shown (n = 3), *p < 0.05, **p < 0.01, ***p < 0.001 versus control, ^#^p< 0.05, ^##^p < 0.01, ^###^p < 0.001 versus CP, ^$^p< 0.05, ^$$^p < 0.01, ^$$$^p < 0.001 versus LY plus CP treatment group.

**Figure 7:**
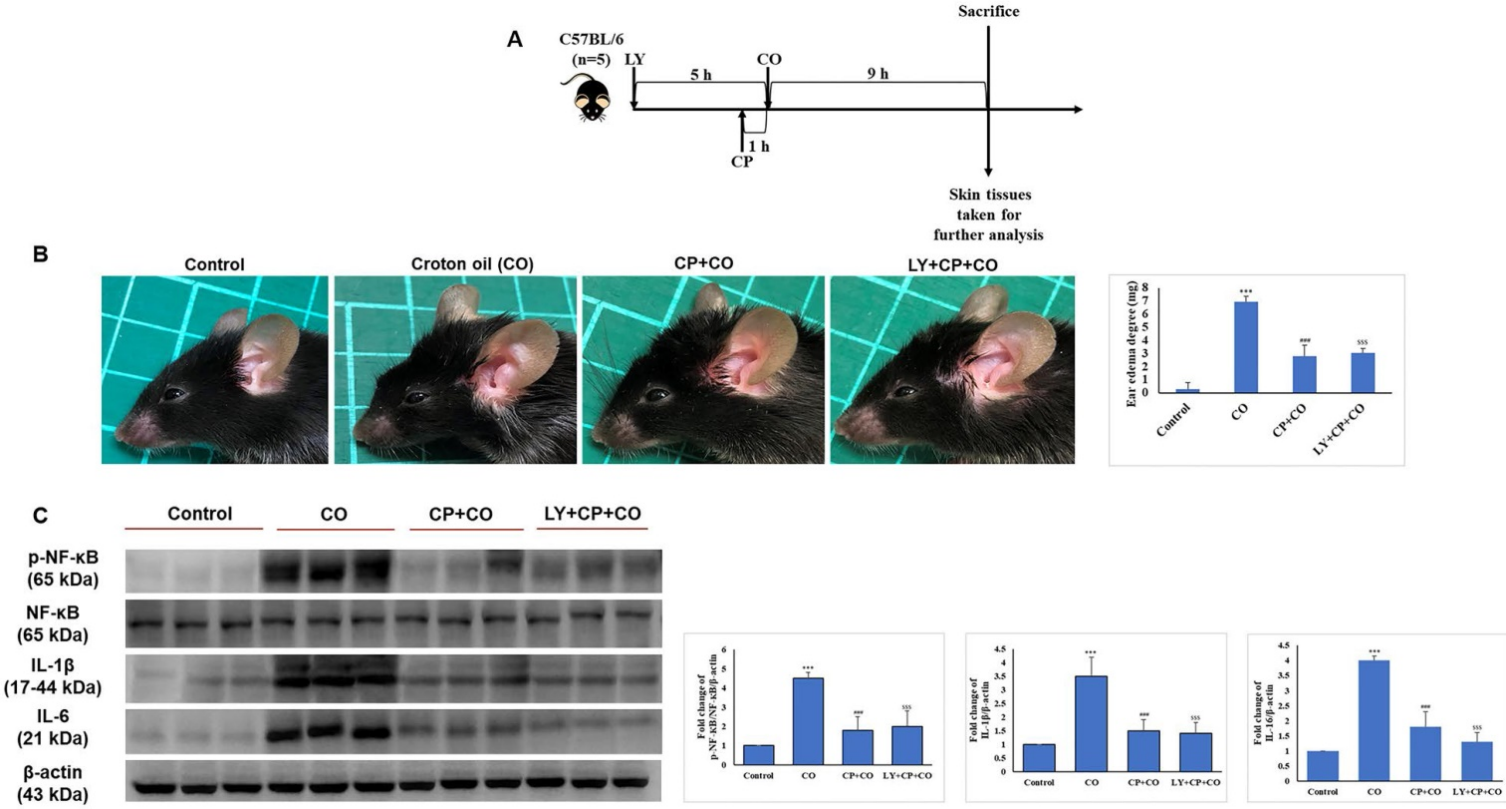
LY treatment maintains the anti-inflammatory activity of GC treatment. **(A)** The detailed animal protocol is described in the material and method section. Croton oil was used to induce ear edema following the treatments of solvents, CP, or CP + LY. **(B)** Ear punch weight (inflammation readout) is presented as fold change versus control. **(C)** The expressions of inflammation (p-NF-kB, NF-kB, IL-6, IL-1β) were evaluated by western blot analysis. Values shown are means ± SE. Quantification of the results is shown (n = 3), *p < 0.05, **p < 0.01, ***p < 0.001 versus control, ^#^p< 0.05, ^##^p < 0.01, ^###^p < 0.001 versus CO, ^$^p< 0.05, ^$$^p < 0.01, ^$$$^p < 0.001 versus CO plus CP treatment group. β- actin was used as a loading control.

Seven-week-old mice were separated into five groups (n = 5): control, vehicle control, CP, CP+LY, and CP+LY+SB (p38 inhibitor). Mice were topically administered CP (1 µg/mouse), LY (15 µM/mouse), and SB (40 µM/mouse) every other day for four weeks; then, sacrificed. Skin tissues were collected for further analysis. As expected, chronic treatment with CP led to severe cutaneous atrophy. Epidermal thickness was reduced by 52% and dermal cellularity was decreased by 48% compared to the vehicle control (Fig. 6B-C). Remarkably, LY significantly protected the skin from the hypoplastic effects of CP. LY+CP-treated skin displayed only 10% and 12% reductions in epidermal and dermal thickness, respectively. However, when p38 was inhibited, the protective effect of LY diminished, resulting in severe atrophy of 56% and 52% in the epidermal and dermal layers, respectively (Fig. 6B-C). Furthermore, protein expression related to the atrophy marker and collagen synthesis signaling pathway showed similar results in the *in vitro* model (Fig. 6D-E). To evaluate the anti-inflammatory effect of CP combined with LY, mouse ears were treated with LY (50 µM), followed by treatment with the contact irritant croton oil (CO) and CP (1 μg/ear). CO-induced inflammation and ear swelling, and CP completely blocked this effect (Fig. 7). Pretreatment with LY did not affect either CO-induced inflammation or the anti-inflammatory effects of CP.

## Discussion

GC-induced skin atrophy is a severe clinical complication devitalizing dermatological barrier function. The adverse effects caused by chronic treatment or high doses of GCs have been hypothesized to involve GR ligands. Therefore, a number of selective GR receptor agonists have been developed and investigated in academic pilot studies, but the outcomes of these studies are still limited 26. Several PI3K/mTOR/Akt inhibitor candidates, including LY, WM, and rapamycin, which are generally known anti-cancer drugs and immunosuppressors, can prevent GR phosphorylation at Ser211, thereby decreasing GR translocation into nucleus 27. However, the mechanism of their applications in dermatology is limited. In our current study, we investigated the advantages and possible mechanism to make GR-targeted therapies safer via the combination of GCs with LY. In particular, the selected dose of LY reversed senescence and collagen synthesis reduction in human dermal fibroblasts (HDFs) after treatment with CP.

FKBP51 is a molecular chaperone that binds GR/Hsp90 heterocomplexes and induces GR affinity for ligands to enhance the nuclear translocation of receptor complexes under stimulation of GCs. In addition to its function as a co-chaperone of the GR complex, FKPB51 also negatively regulates signaling pathways, such as Akt and mTOR, which play a vital role in cell progression 28. In addition, several studies related to the skin have demonstrated the upregulation of phosphorylated GR and FKBP51 together under GCs exposure 29. FKBP51 was identified as an atrophic gene. In mice knocked-out FKBP51, all skin cells and interfollicular epidermal stem cells were protected against GC-induced atrophy. It has been reported that the levels of phosphorylation of AktSer473, mTORSer2448, and pro-proliferative effectors, S6K1 and rpS6, were preserved in human keratinocytes blocking FKBP51 and in the dorsal skin of FKBP51 knock-out mice 9.

Recently, a change was observed in the intracellular localization and function of GR in human keratinocytes and mouse skin after FKBP51 was suppressed by PI3K inhibitors 10. It was shown previously that the combination of PI3K inhibitors and GCs not only suppress FKBP51 expression resulting in reduction of GC-induced atrophy in keratinocytes, but do so whilst maintaining the anti-inflammatory activity of GCs 10. Nonetheless, GC-induced skin atrophy reported in the clinical setting not only occurs in keratinocytes of the epidermis but also impairs the physiological progression of fibroblasts in the dermis, such as proliferation, senescence, and collagen synthesis. In light of these data, the efficiency of the selected dose of LY was experimentally validated to suppress FKBP51 expression at the protein level *in vitro* in HDFs and *in vivo* in mouse skin (Fig. 3B, 6C-D). The results showed that both senescence and collagen synthesis reduction were improved by treatment with a combination of LY and CP (Fig. 3B, 6C-D).

Furthermore, LY strongly modified GR activity, shifting it towards trans-repressive signaling. To modulate GR activity, LY acted as an inhibitor of GC involvement in the TGF-β/Smad signaling (Fig. 3A). Impairment of TGF-β activation suppresses p-Smad2/3 to complete its function of positively regulating collagen synthesis. Several studies have demonstrated that GR regulates TGF-β1 signaling by specifically inhibiting Smad3 and Smad4 activation in a ligand-dependent manner in nucleus, resulting in a decrease in collagen synthesis. In our current study, treatment with LY inhibited GR translocation, resulting in the stability of the transcription factor of collagen synthesis in nucleus (Fig. 4A-C), and in turn, it reserved collagen (Fig. 3B, 6C-D) against the side effects of CP. However, despite the blockage of trans-repression of GCs, which has the potential for NF-κB suppression, LY maintained the anti-inflammatory effect of CP via inhibition of NF-κB and inflammatory cytokines in the cytoplasm (Fig. 4D, Fig. 7).

Interestingly, we demonstrated that p38 MAPK was involved in the protective effect of LY to regulate the collagen synthesis pathway and GR activation. Under GC stimulation, GR requires the presence of co-regulatory proteins comprising Hsp90 and FKBP immunophilins for phosphorylation and efficient nuclear import 26. GR is phosphorylated at multiple Ser and Thr residues at the N-terminus by MAPKs, cyclin-dependent kinases (CDKs), casein kinase II, and glycogen synthase kinase-3β (GSK-3β), and its phosphorylation status defines its nuclear import and function 39. Several studies have demonstrated that p38 MAPK negatively regulates GR phosphorylation and nuclear translocation following GC exposure. Meanwhile, p38 MAPK also plays a positive role in dermal collagen synthesis 16, 18, 19. In this context, our results showed that CP significantly decreased the activation of MAPK proteins in a dose- and time-dependent manner (Fig. 1D). Inhibitors of p38, ERK, and JNK were shown to reverse the inhibitory effect of LY treatment mediated by p38 in a dose-dependent manner under CP exposure (Fig. 3D, Fig. 5B), indicating that LY inhibited GC-induced skin atrophy through p38 activation (Fig. 5).

In addition to the inhibition of FKBP51 and GR modulation, among other PI3K inhibitors, LY has the same effect as rapamycin to inhibit mTOR (Fig. 2C), which has been reported to improve senescence. It has been recently increasing evidences of PI3K/PDK1/Akt signaling-induced senescence in various cell types including human dermal fibroblasts 30-32. This phenomenon could be explained that at physiological levels, Akt signaling leads to the activation of MDM2 at levels that maintain low p53 protein expression, but chronic hyperactivation of Akt results in mTORC1-dependent increases in p53 translation and simultaneously stimulates MDM2 sequestration within nucleus. Although the mTOR pathway is one of the fundamental signaling pathways for cell growth and progression, when cell cycle is abruptly arrested by the aging markers p16 or p21, the mTOR upregulation results in growth-like conversion from reversible arrest (quiescence) to senescence, indicating that mTOR activates geroconversion 33. Rapamycin and its analogs, as well as p-mTOR inhibitors, suppress geroconversion, thereby maintaining cells in a healthy young state. Moreover, these drugs prevent abnormal proliferation of aged cells and restrict senescence 33. The anti-senescence effect of rapamycin has been demonstrated in various *in vivo* and *in vitro* studies 34, 35. Therefore, inhibition of PI3K/PDK1/Akt/mTOR can revert cellular senescence.

Furthermore, it can be explained that the keys of different application of PI3K inhibitors between anti-cancer and cellular protection are dependent on dose and time treatment. For anti-cancer, LY alone treatment requires high doses (25-75 µM) for 24-48 h 36. Unexpectedly, Lee's report also indicated that LY alone treatment did not decrease cell survival in human cervical cancer cell lines 37. In contract, for cellular protection, PI3K inhibitors are usually applied as pretreatment in short time without affecting proliferation. For examples, 20 µM of resveratrol, another PI3K inhibitor 38, for 1 h suppressed UVB-induced MMP1 expression and collagen synthesis decrease in human dermal fibroblasts 39, 20 - 40 µM quercetin, another PI3K inhibitor 40, for 1h directly targeted JAK2 and PKCδ to prevent UV-induced photoaging in human skin 41. In the present study as shown in Fig. 2, high dose of LY at 50 µM can reduce HDFs viability and collagen synthesis; however, the selected dose at 15 µM for the entire study showed PI3K/Akt signaling suppression only at short time at 6 h, but back to normal at 24 h without growth inhibition and collagen synthesis decrease. In addition, we also performed 20 µM resveratrol as positive control (Supplementary Fig. 1). Resveratrol was also able to protect collagen synthesis and cell prime from glucocorticoid. However, the FKBP51 and p-GR, the markers of skin atrophy enhanced by GC did not changed by resveratrol combination treatment, indicating resveratrol exerted its normal function but did not show special beneficial reaction on GC-induced skin diseases. These findings indicate that the action of LY at low dose regarding a protective role could be different from that at high dose as senescence enhancing role. Based on our data, pretreatment with LY inhibited mTOR during glucocorticoid intervention in HDFs and suppressed the expression of aging markers such as β-gal (Fig. 3C). Thus, we believe that LY has the same pathway as rapamycin in reducing skin senescence.

## Conclusions

In brief, our study provides evidence for the protective effect of LY against GC-induced skin atrophy in human dermal fibroblasts. This process involves the inhibition of PI3K/AKT/mTOR signaling to control senescence in fibroblasts, the suppression of the atrophy marker FKBP51, and the inhibition of GR activation, which negatively regulates the transcription factor of collagen synthesis via p38 MAPK.

## Supplementary Material

Supplementary figures.

## Abbreviations

17-DMAG: Heat shock protein 90 inhibitor
AP-1: activator protein 1
BSA: bovine serum albumin
CDK: cyclin-dependent protein kinases
CHIP: chromatin immunoprecipitation
CO: croton oil
COL1A1: collagen type 1
COL3A1: collagen type 3
CP: clobetasol propionate
DMEM: Dulbecco's modified eagle media
ECM: extracellular matrix
EVG: Verhoeff-van Gieson's staining
FBS: fetal bovine serum
FKBP51: FK506 binding protein 5
GC: glucocorticoid
GR: glucocorticoid receptor
GRE: glucocorticoid-responsive elements
H&E: hematoxylin and eosin staining
Hsp90: heat shock protein 90
HDFs: human dermal fibroblasts
IHC: immunohistochemistry
IL-6: interleukin 6
IL-1β: interleukin 1β
JNK: c-jun N-terminal kinase
LY: LY294002
M&T: masson's trichrome staining
MAPK: mammalian MAP kinase
MMP: matrix metalloproteinase
NF-КB: nuclear factor kappa-light-chain-enhancer of activated B cells
OTC: over-the-counter
PBS: phosphate buffer saline
PD: PD98059-ERK inhibitor
PHLPP1/2: protein kinase C quality control by phosphatase 1 and 2
PI3K: phosphoinositide 3-kinase
PVDF: polyvinylidene difluoride
qPCR: quantitative PCR
ROS: reactive oxygen
SA-β-gal: β-galactosidase staining
SAFit2: FKBP51 inhibitor
SBE: Smad-binding elements
SB: SB203580-p38 MAPK inhibitor
SD: standard deviation
SP: SP600125-JNK inhibitor
TEWL: trans-epidermal water loss
TGF-β: transforming growth factor beta
TGFβR1: TGF-β receptor I
TGFβR2: TGF-β receptor II
UV: ultraviolet radiation
