# Supplementary material for: Reversion of glucocorticoid-induced senescence and collagen synthesis decrease by LY294002 is mediated through p38 in skin
Source: Int J Biol Sci. 2022 Oct 18;18(16):6102–13. doi: 10.7150/ijbs.73915 (PMC9682531; doi:10.7150/ijbs.73915)
Supplement: Supplementary file 1 — Supplementary figures. [file ijbsv18p6102s1.pdf]

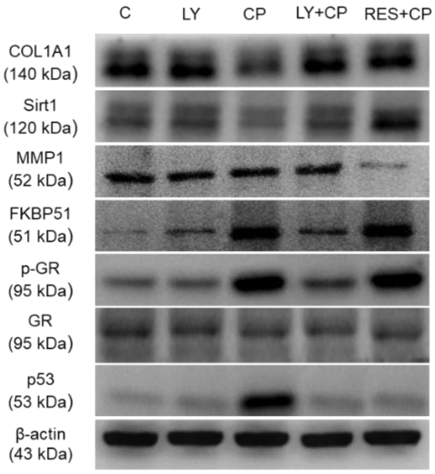

Supplementary Fig. 1. The protective effects of LY and resveratrol on GC-induced senescence and collagen synthesis decrease in HDFs. Cells were pretreated with 15 μM LY or 20 μM resveratrol for 6 h, then treated with 0.1 μM CP for 24 h. The expressions of COL1A1, Sirt1, MMP1, FKBP51, p53, GR and p-GR in HDFs were identified by western blot. β-actin was used as a loading control.

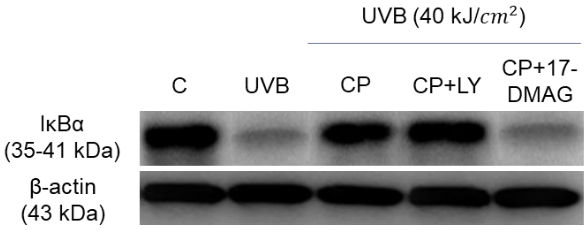

Supplementary Fig. 2. The selected dose of LY maintains the anti-inflammatory action of GC in cytoplasm. Cells were pre-treated with 15 μM LY or 10 μM 17-DMAG for 6 h, then treated with 0.1 μM CP for 24 h. The expression of IκBα was evaluated by western blot analysis. β-actin was used as a loading control.

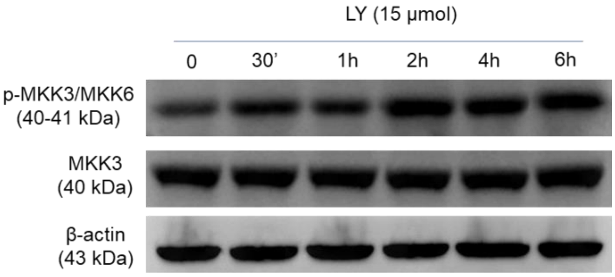

Supplementary Fig. 3. LY enhances the activation of p38 in a time-dependent manner. Cells were treated with 15 μM LY for indicated times. The levels of p-MKK3/MKK6 and MMK3, the upstream of p38, were evaluated by western blot analysis. β-actin was used as a loading control.
